# The *Salmonella enterica* PhoP Directly Activates the Horizontally Acquired SPI-2 Gene *sseL* and Is Functionally Different from a *S. bongori* Ortholog

**DOI:** 10.1371/journal.pone.0020024

**Published:** 2011-05-19

**Authors:** Ohad Gal-Mor, Dana Elhadad, Wanyin Deng, Galia Rahav, Brett B. Finlay

**Affiliations:** 1 Infectious Diseases Research Laboratory, Sheba Medical Center Tel-Hashomer, Tel-Hashomer, Israel; 2 Michael Smith Laboratories, University of British Columbia, Vancouver, British Columbia, Canada; 3 Sackler Faculty of Medicine, Tel Aviv University, Tel-Aviv, Israel; Indian Institute of Science, India

## Abstract

To establish a successful infection within the host, a pathogen must closely regulate multiple virulence traits to ensure their accurate temporal and spatial expression. As a highly adapted intracellular pathogen, *Salmonella enterica* has acquired during its evolution various virulence genes via numerous lateral transfer events, including the acquisition of the *Salmonella* Pathogenicity Island 2 (SPI-2) and its associated effectors. Beneficial use of horizontally acquired genes requires that their expression is effectively coordinated with the already existing virulence programs and the regulatory set-up in the bacterium. As an example for such a mechanism, we show here that the ancestral PhoPQ system of *Salmonella enterica* is able to regulate directly the SPI-2 effector gene *sseL* (encoding a secreted deubiquitinase) in an SsrB-independent manner and that PhoP plays a part in a feed-forward regulatory loop, which fine-tunes the cellular level of SseL. Additionally, we demonstrate the presence of conserved *cis* regulatory elements in the promoter region of *sseL* and show direct binding of purified PhoP to this region. Interestingly, in contrast to the *S. enterica* PhoP, an ortholog regulator from a *S. bongori* SARC 12 strain was found to be impaired in promoting transcription of *sseL* and other genes from the PhoP regulon. These findings have led to the identification of a previously uncharacterized residue in the DNA-binding domain of PhoP, which is required for the transcriptional activation of PhoP regulated genes in *Salmonella* spp. Collectively our data demonstrate an interesting interface between the acquired SsrB regulon and the ancestral PhoPQ regulatory circuit, provide novel insights into the function of PhoP, and highlight a mechanism of regulatory integration of horizontally acquired genes into the virulence network of *Salmonella enterica*.

## Introduction

*Salmonella* spp. infects various animal and human hosts and is a major cause of diverse diseases including enteric fever, gastroenteritis, bacteremia, and systemic infections worldwide. Current taxonomy of the genus *Salmonella* includes the species *S. enterica* with a large number of clinically important serovars that infect animal and human hosts and *S. bongori*, which represents a phylogenetically older lineage of the *Salmonella* genus and is mainly associated with cold-blooded vertebrates [1].

As with other pathogens, lateral gene transfer plays a key role in the evolution of *Salmonella* in adaptation to changes in the environment and exploitation of new niches [2]. Horizontal acquisition of mobile genetic elements has been shown to provide a wealth of genetic diversity and a source of various virulence factors required during the infection. In some cases, the acquired elements consist of large virulence gene clusters, designated *Salmonella* pathogenicity islands (SPIs) such as SPI-1 and SPI-2. The acquisition of these SPIs into the bacterial genome is considered to be a ‘quantum leap’ in *Salmonella* evolution [3]. SPI-1 is present in both *Salmonella* species and required for *Salmonella* invasion into the intestinal epithelium and non-phagocytic cells [4]. SPI-2 is found in *S. enterica* species, but not in *S. bongori*, and represents a second, more recent event in *Salmonella* evolution [5]. SPI-2 is essential for intracellular survival and replication and has a crucial role in systemic infections in mammals [6], [7]. Both SPI-1 and SPI-2 encode a separate type three secretion system (T3SS) used to deliver effector proteins directly into the cellular environment of the eukaryotic host, manipulating various host pathways (reviewed in [8]).

SseL is a SPI-2 translocated effector encoded outside of the SPI-2 locus and, like other SPI-2 effectors, is absent from the *S. bongori* genome [9]. A *S. enterica* serovar Typhimurium (*S.* Typhimurium) *sseL* mutant strain is attenuated for virulence in mice [9], [10] and SseL functions as a deubiquitinase [10] that modulates host inflammatory responses by suppressing NF-κB activation and IκBα ubiquitination and degradation [11].

Bacterial pathogenicity is a multifactorial trait that requires appropriate expression of numerous virulence genes. Fine-tuning of bacterial virulence factor expression is achieved by a synchronized operation of regulatory pathways in response to environmental cues. Among others, *S. enterica* utilizes two pivotal two-component regulatory systems known as PhoPQ and SsrAB to control the expression of genes required for its intracellular life-style.

PhoQ is a sensor for extracellular Mg^2+^ that modifies the phosphorylation state of the DNA-binding protein PhoP [12], [13]. PhoP belongs to the OmpR family of winged-helix transcription factors (reviewed in [14]) and controls the expression of a large number of genes that mediate adaptation to low Mg^2+^ environments and/or virulence in several Gram-negative species including *Salmonella enterica* and *Escherichia coli* [15]. It is believed that by monitoring extracellular Mg^2+^, PhoPQ allows *Salmonella* to sense the transition from an extracellular environment to a subcellular location and to activate a set of virulence factors, which are required for intracellular infection [16]. Indeed, *S.* Typhimurium strains lacking the PhoPQ system are highly attenuated for virulence in mice and unable to proliferate within macrophages [16], [17].

While the PhoPQ pathway is conserved among *Salmonella* and related species (PhoP and PhoQ of *E. coli* and *S. enterica* are 93% and 86% identical, respectively [18]) and considered as an ancestral regulatory system, the SPI-2 encoded SsrAB system is unique to *S. enterica*. SsrAB is composed of the histidine kinase sensor, SsrA, and the response regulator, SsrB, a member of the NarL/FixJ subfamily. The expression of SPI-2 genes and SPI-2 T3SS-associated effectors located outside of SPI-2 is induced within host cells and is strictly dependent on the SsrAB system (reviewed in [19]). In addition, expression of the SsrAB regulon members was shown to be positively affected by the two-component system EnvZ-OmpR [20], [21], the transcriptional regulator SlyA [22], [23], and several nucleoid-associated proteins such as the integration host factor (IHF) and the factor for inversion stimulation (Fis; reviewed in [24]). Interestingly, an epistatic interaction between PhoP and the SsrAB system has been demonstrated by the ability of PhoP to bind the *ssrB* promoter and regulate SsrA post-transcriptionally [25].

Here we characterized the role of PhoP in governing the expression of the SPI-2 effector gene, *sseL*. We demonstrate a regulatory integration of a horizontally acquired virulence gene into the ancestral PhoPQ networks and show that PhoP directly controls *sseL* expression in a feed-forward regulatory loop. Moreover, we demonstrate that a *S. bongori* SARC 12 strain is impaired in activation of the PhoP regulon due to a single amino acid substitution in the C-terminal DNA binding domain of PhoP.

## Results

### PhoP facilitates *sseL* transcription in an SsrB-independent manner

A differential fluorescence induction (DFI) screen of a *gfp* reporter-gene library was performed to identify *S.* Typhimurium genes, which are induced under a defined set of conditions (low Mg^+2^, low phosphate, acidic pH) assumed to mimic the intracellular milieu (our unpublished results). In the final pool of 93 sequenced clones we found a significant representation of various known PhoP regulated genes including *pagO* (5 times) [26]; *mig*-5 (also known as PSLT046; 2 times) [27]; *pmrD* (2 times) [28]; and *ybjX* [29], together with a dominant presence of *sseL*, which was hit 8 times. A previous study showed that SsrB is absolutely required for *sseL* expression and that it is induced inside macrophages [9]. Nonetheless, co-isolating PhoP regulon members with *sseL* under the same enrichment protocol prompted us to look more closely on the potential role of PhoP as a regulator that controls *sseL* expression in conjunction with SsrB.

**Figure 1:**
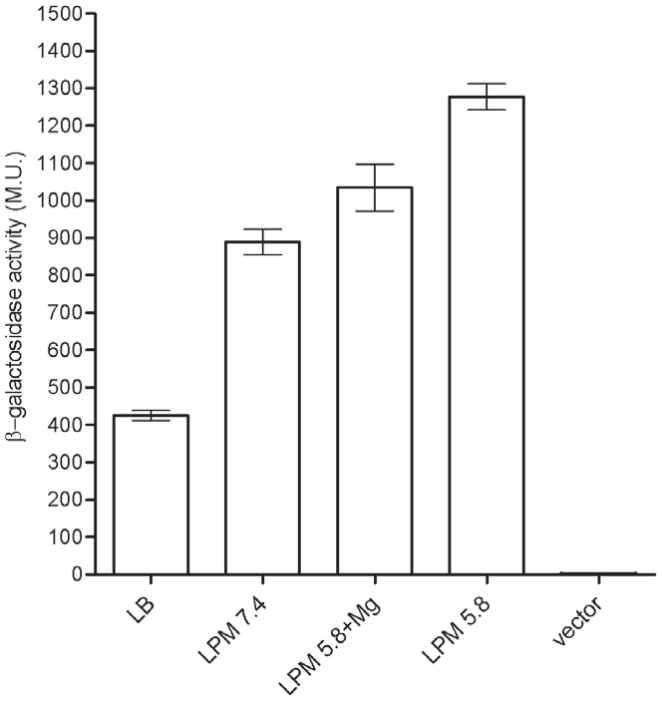
*sseL::lacZ* is induced in response to low phosphate, low magnesium and acidic pH environmental cues. *S.* Typhimurium strains carrying *sseL:lacZ* were grown for 16 h at 37°C in LB, LPM (pH 7.4) LPM (pH 5.8) supplemented with 10 mM MgCl_2_, and LPM (pH 5.8), and were assayed for β-galactosidase activity presented in Miller units (M.U.). Basal *lacZ* expression of *S.* Typhimurium harboring pMC1403 (vector) that was grown in LPM (pH 5.8) is also shown.

To elucidate the involvement of PhoP in the regulation of *sseL*, a reporter-gene fusion between *sseL* and a promoterless β-galactosidase was constructed using the vector pMC1403 [30]. Comparison of *sseL::lacZ* expression under several growth conditions showed the contribution of minimal medium, acidic pH, and phosphate/magnesium starvation cues to the induction of *sseL* and indicated that maximal expression was reached during the stationary phase in defined low phosphate, low magnesium minimal (LPM) medium (Fig. 1). These results closely matched the previously described high protein levels of SseL-HA [9] and induction of other SsrB regulated genes [31], [32], [33] under similar conditions, and also demonstrated the capability of our experimental system to correctly report different levels of *sseL* expression.

**Figure 2:**
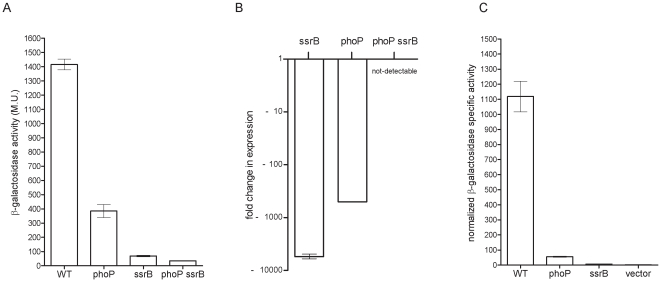
PhoP activates the expression of *sseL* in an SsrB-independent manner. (A) β-galactosidase expression of *S.* Typhimurium wild-type (WT), *phoP*, *ssrB* and *phoP ssrB* double mutant strains harboring *sseL::lacZ* grown in LPM medium (pH 5.8). (B) Expression of *sseL* transcripts in *ssrB*, *phoP*, and *phoP ssrB* double mutant backgrounds vs. the wild-type strain as determined by q-RT-PCR. RNA was harvested from *Salmonella* strains grown in LPM medium to late logarithmic phase. The purified RNA was reverse-transcribed and the expression of *sseL* was examined by quantitative real-time PCR. The fold change in the abundance of *sseL* transcripts in the different mutants relative to their expression in the wild-type strain is presented. Expression was normalized using the housekeeping *rpoD* gene as a control. The results represent the mean of 8 RT-PCR reactions from two independent RNA preparations with a standard error shown by the error bars. (C) Expression of *sseL::lacZ* during macrophages infection. *S.* Typhimurium wild-type (WT) harboring *sseL::lacZ* or the vector (pMC1403) only and *phoP*, *ssrB* isogenic mutant strains carrying *sseL::lacZ* were used to infect J774.1 macrophages. Six hours post-infection, the cells were harvested; β-galactosidase activity was measured and normalized by the intracellular CFU count. The values are presented in normalized Miller units and are the mean of four independent infected cultures.

Subsequently, we were interested in assessing the relative contribution of PhoP and SsrB to the integrated regulation of *sseL*. Examining the expression levels of *sseL::lacZ* in the *ssrB* and *phoP S.* Typhimurium mutant strains showed a significant (*P*<0.0001) decrease by ∼20, and 4-fold, respectively, in comparison to its expression in the wild-type (Fig. 2A). These data highlighted the fundamental role of SsrB and indicated that PhoP is also involved in the *sseL* regulatory network, directly or indirectly.

To better understand the mechanism by which PhoP facilitates *sseL* expression, we explored the expression of *sseL::lacZ* in a *phoP ssrB* (OG2011) double mutant strain. The rationale behind this experiment was the assumption that if PhoP contributes to the expression in an SsrB-dependant manner, i.e. functioning upstream of SsrB in the same regulatory cascade, the expression in the double mutant strain is likely to be similar to the expression in the *ssrB* background; however, if the contribution by PhoP is independent of SsrB, then an accumulative effect is expected, leading to further reduced expression in the double mutant background. *sseL::lacZ* expression in the *phoP ssrB* double mutant showed ∼2-fold lower expression compared to that in the *ssrB* strain (*P*<0.0001) supporting the possibility that PhoP and SsrB have accumulative effects on the expression of *sseL*, and that PhoP contributes to the expression of *sseL* by an SsrB-independent manner, in addition to its epistatic regulation of *ssrB*. To further test this hypothesis, we conducted quantitative reverse transcription PCR (qRT-PCR) determining directly the mRNA levels of *sseL* transcripts in the wild-type, *ssrB*, *phoP*, and *phoP ssrB* backgrounds. RNA harvested from *Salmonella* cultures that were grown in LPM medium showed a prominent reduction of ∼5600 and 500-fold in the abundance of *sseL* transcripts in the *ssrB* and *phoP* backgrounds, respectively, relative to the wild-type strain. In the *phoP ssrB* double mutant strain, the transcription of *sseL* was further reduced to levels that were below the detection threshold of the RT-PCR (Fig. 2B). These data provided further evidence that PhoP can contribute to *sseL* transcription in an SsrB-independent mechanism.

### PhoP is required for *sseL* expression during intracellular infection

*sseL* was shown to be readily expressed inside macrophages [9], [10] and therefore, we were interested in characterizing the role of PhoP in *sseL* induction during macrophage infection. J774.1 macrophages were infected with wild-type, *phoP* and *ssrB S.* Typhimurium strains harboring *sseL::lacZ*, or the vector only. At six hours post-infection, the cells were harvested, and the intracellular expression of the reporter strains, as well as their intracellular survival, was determined. As shown in Fig. 2C, a remarkable reduction was observed in the normalized intracellular expression of *sseL::lacZ*, when macrophages were infected with *phoP* or *ssrB* mutant strains. *sseL::lacZ* expression results in the intracellular bacteria were correlated with the *in-vitro* analyses and showed that in addition to SsrB, PhoP is required for maximal *sseL* expression in the intramacrophage environment.

### *sseL* promoter is conserved among *S. enterica* subspecies I and harbors two putative PhoP boxes

**Figure 3:**
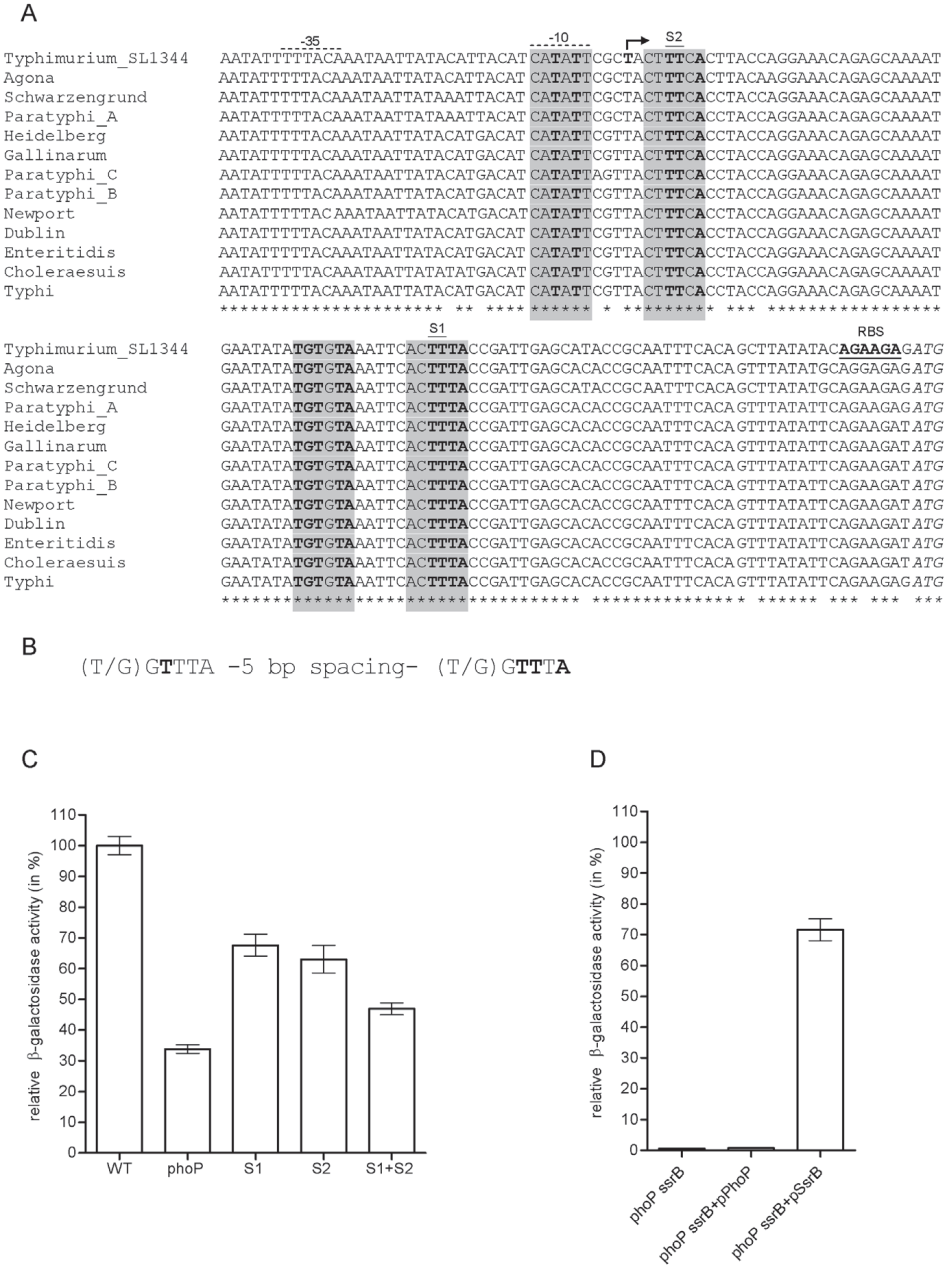
The regulatory region of *sseL* is highly conserved and possesses two putative PhoP binding sites. (A) Sequence alignment of 137-bp upstream from the predicted start codon of SseL (GI:267994379) is shown for *S.* Typhimurium SL1344 (FQ312003.1); *S.* Agona (CP001138.1); *S.* Schwarzengrund (CP001127.1); *S.* Paratyphi A (FM200053.1); *S.* Heidelberg (CP001120.1); *S.* Gallinarum (AM933173.1); *S.* Paratyphi C (CP000857.1); *S.* Paratyphi B (CP000886.1); *S.* Newport (CP001113.1); *S.* Dublin (CP001144.1); *S.* Enteritidis (AM933172.1); *S.* Choleraesuis (AE017220.1); and *S.* Typhi (AE014613.1). The identified transcription start site in *S.* Typhimurium is indicated in bold and by the bent arrow. The predicted ribosomal binding site (RBS) is shown in bold and underlined; the predicted start codon of *sseL*, (*ATG*), is in italics; and the probable σ^70^ −35 and −10 elements are marked by broken upper-line. The putative PhoP binding sites are indicated by shaded boxes and the nucleotide residues that match the canonical PhoP-box are in bold. S1 and S2 indicate the two conserved thymines (in the second hexanucleotide at positions 3 and 4) that were replaced by site directed mutagenesis. (B) Nucleotide architecture of a canonical PhoP-box. Conserved positions that are necessary and sufficient for DNA binding by PhoP are marked in bold. (C) Relative expression (from the wild-type) of *sseL::lacZ* is shown for a wild-type promoter in *S.* Typhimurium (WT), a wild-type promoter in a *phoP* mutant background (phoP), and manipulated promoters harboring S1, S2 or S1 and S2 nucleotide substitution (from TT to GG) in the predicted PhoP binding sites (as indicated in A) in a wild-type background. (D) Relative expression (from the wild-type) of *sseL::lacZ* driven by a modified promoter (harboring mutation in both S1 and S2 sites) is shown in a *S.* Typhimurium *phoP ssrB* background without complementation (*phoP ssrB*), in the presence of *S.* Typhimurium PhoP (*phoP ssrB*+pPhoP), or SsrB (*phoP ssrB*+pSsrB). All *S.* Typhimurium cultures were grown in LPM medium to late logarithmic phase (6 h).

As opposed to some SPI-2 effector genes with only limited serovar distribution, *sseL* is present in many different serotypes of *S. enterica* subspecies I. Comparison of the regulatory region of *sseL* in 13 *S. enterica* serovars (Fig. 3A) showed a very high degree of promoter conservation, including in serovars that are either host-restricted (Typhi and Paratyphi to human and Gallinarum to poultry) or host-adaptive (Dublin to cattle and Choleraesuis to swine). These highly conserved promoters might reflect the outcome of a selective pressure against genetic changes in this locus and suggest that *sseL* is similarly regulated by different serovars and within different hosts.

To identify putative *cis* regulatory elements in the context of a functional promoter, we determined the transcription start site of *sseL* in *S.* Typhimurium by an RNA ligase mediated (RLM) rapid amplification of cDNA ends (RACE). This analysis identified a transcriptional initiation site corresponding to a T residue located 97-bp upstream to the predicted start codon of SseL (GI:267994379; or 28-bp upstream from a differently predicted start codon according to GI:308065958). In the sequences upstream of the transcription initiation site, probable σ^70^ −35 ( **TT**T**ACA**) **and −10 (** **C**ATA**T**T) elements separated by 18 nucleotides were also identified (Fig. 3A). These elements are in agreement with the consensus sequences TTGACA (−35) and TATAAT (−10) [34] and fit the preferred spacing range of 15–19-bp between the −35 and −10 elements [35].

The results suggesting that *sseL* is activated by PhoP have led us to search for the presence of potential PhoP binding sites. A conserved repeat of the hexanucleotide (T/G)GTTTA separated by 5 nucleotides, known as PhoP box (Fig. 3B), is often present in the promoter region of PhoP-regulated genes in *Salmonella* and in *E. coli* [29], [36]. A conserved thymine in the first hexanucleotide (at position 3) and two conserved thymines together with one conserved adenine in the second hexanucleotide (at positions 3, 4, and 6, respectively) were necessary and sufficient for DNA binding of PhoP in *in-vitro* DNA footprinting assays [37], [38], [39], [40]. The *sseL* promoter region was found to harbor two putative sites resembling the characterized PhoP binding sequences as shown in Fig. 3A. Interestingly, one putative PhoP box was found downstream from the transcription start site (+1) of *sseL*, while the other overlapped with the transcription start site and the −10 element.

In order to examine whether these sites contribute to the expression of *sseL*, the two conserved thymines in the second hexanucleotide of each site were replaced with guanine residues. As illustrated in Fig. 3C, site directed mutagenesis of either site 1 (S1) or site 2 (S2) resulted in a moderate but significant (*P*<0.0001) decrease in the expression of *sseL::lacZ*. Additionally, an *sseL* manipulated promoter containing both mutations (S1+S2) demonstrated accumulative reduction in the expression of *sseL::lacZ* by more than 2-fold (*P*<0.0001), indicating that the S1 and S2 sites are required for optimal expression of *sseL*, possibly due to their role as PhoP binding sites. To further examine this idea and to rule out the possibility that these positions are used as SsrB binding sites, we analyzed the expression of an altered *sseL* promoter harboring both mutations in a *phoP ssrB* background complemented with PhoP or SsrB, provided *in trans*. In the presence of SsrB, this mutated promoter was readily able to induce *sseL::lacZ* expression, suggesting that SsrB does not bind to these sites. In contrast, providing PhoP *in trans* did not induce *sseL::lacZ* expression from the modified promoter (Fig. 3D), supporting the function of S1 and S2 loci as potential PhoP binding sites.

### PhoP binds directly to the promoter region of *sseL*

**Figure 4:**
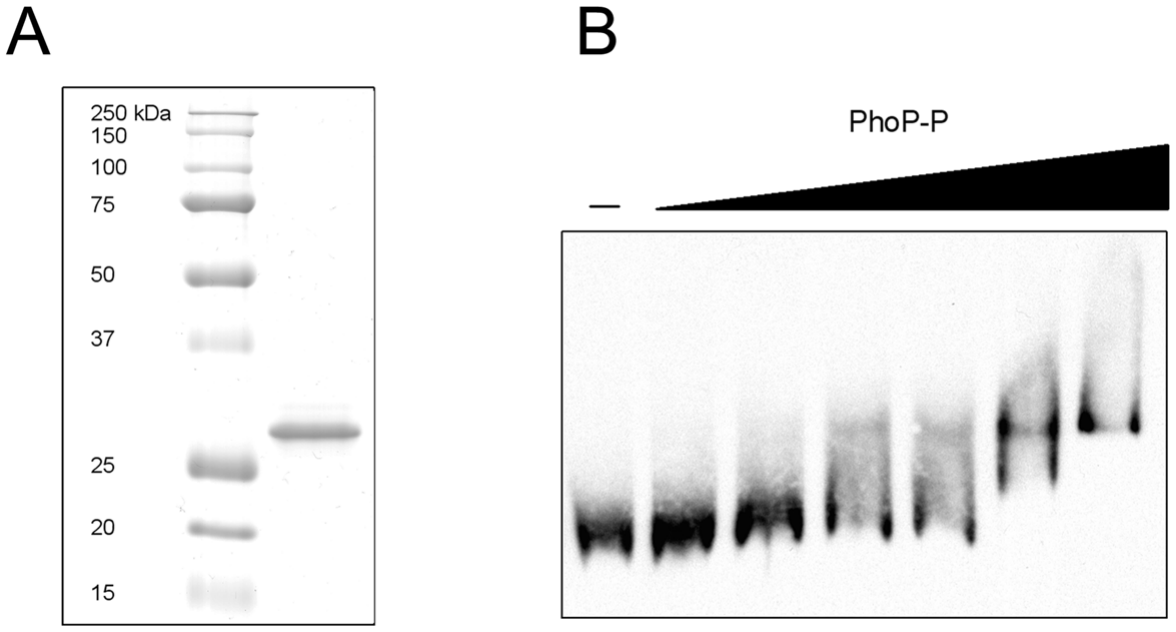
His-PhoP binds to the promoter regions of *sseL*. (A) 5 µg of the purified His-tagged PhoP protein was analyzed on an SDS-PAGE by Coomassie Blue staining to assess its purity. Molecular weight markers (kDa) are shown on the left. (B) Electrophoretic mobility shift assay analysis of *sseL* promoter. A 128-bp DNA fragment containing the putative PhoP binding sites of the *sseL* promoter was Dig-labeled. 15 fmol of the labeled probe was incubated at 37°C in a binding buffer containing a 100-fold excess of dI-dC as a nonspecific DNA competitor, in the presence of increasing amounts of phosphorylated His-PhoP protein (0, 12.5, 25, 50, 100, 200, 400 pmol). The PhoP-DNA mixtures were subjected to a 6% native polyacrylamide gel electrophoresis and imaged as described in Materials and Methods.

The above results implied that PhoP might interact directly with *sseL* to activate its expression. To investigate the possibility of direct binding of PhoP to the promoter region of *sseL*, we conducted an electrophoretic mobility shift assay (EMSA) using a *S.* Typhimurium N-terminally His-tagged PhoP protein (His-PhoP). In this analysis, purified His-PhoP protein (Fig. 4A) was incubated for 30 min at 37°C with a 128-bp dUTP-digoxigenin labeled DNA fragment corresponding to the predicted regulatory sequence of *sseL*. Subsequently, the DNA-protein complex was resolved on a 6% native polyacrylamide gel and imaged. EMSA analysis showed that His-PhoP alone could gel shift the promoter region of *sseL* in a dose dependent manner and in the presence of a 100-fold excess of a non specific DNA (dI-dC), indicating the ability of PhoP to bind *in-vitro* to the *sseL* promoter (Fig. 4B).

### The *S. enterica* PhoP, but not an impaired ortholog from a *S. bongori* strain, activates *sseL* expression

**Figure 5:**
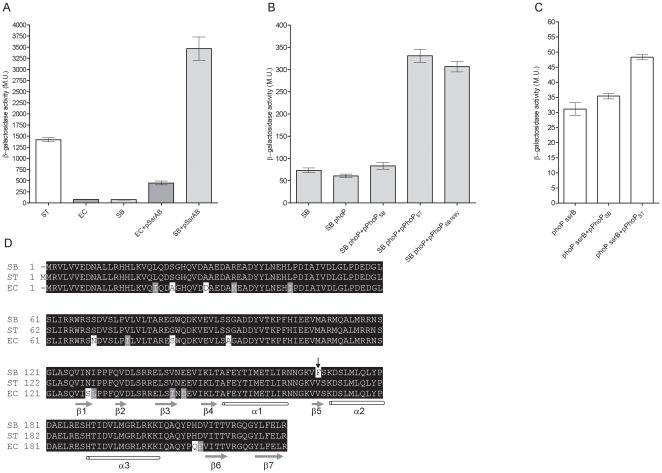
The PhoP regulator from *S. enterica* and a *S. bongori* SARC 12 strain are functionally different. (A) *S.* Typhimurium (ST), *E. coli* MC1061 (EC), *S. bongori* SARC 12 (SB) harboring *sseL::lacZ* in the presence or absence of SsrAB (pSsrAB) were tested for their β-galactosidase activities following growth in LPM medium. (B) *S. bongori* SARC 12 (SB), *S. bongori phoP* (SB phoP), and *S. bongori phoP* strain complemented with a *S. bongori phoPQ* (pPhoP_SB_), *S.* Typhimurium *phoPQ* (pPhoP_ST_), or the *S. bongori phoPQ* harboring V169 (pPhoP_SB169V_) all carrying *sseL::lacZ* were tested for their β-galactosidase activity following growth under inducing conditions. (C) *sseL::lacZ* expression is shown in a *S.* Typhimurium *phoP ssrB* background without complementation (*phoP ssrB*), in the presence of the *S. bongori* PhoP (*phoP ssrB*+pPhoP_SB_) or in the presence of the *S.* Typhimurium PhoP (*phoP ssrB*+pPhoP_ST_). (D) Amino acid alignment of PhoP sequences from *S. bongori* SARC 12 (SB), *S.* Typhimurium SL1344 (ST), and *E. coli* (EC; P23836) is shown. Identical, similar and disparate amino acids are shown in black, grey, and white, respectively. Secondary structural elements (α-helices in white and β-strands in grey) within the C-terminus domain were predicted using the SOPMA program and are correlated with the *E. coli* OmpRc structure. Amino acid variation between *S. bongori* and *S.* Typhimurium at position 169 is indicated by the black arrow.

To further characterize the relative contribution of PhoP and SsrB to the transcriptional output of *sseL* we sought to analyze *sseL::lacZ* expression in two SsrB-free heterologous hosts, *E. coli* and *S. bongori*, representing closely related species that lack the entire SPI-2 locus and its related effectors. Expression of *sseL::lacZ* in *E. coli* and *S. bongori* was found to be ∼18-fold lower relative to the expression in *S.* Typhimurium. We hypothesized that these differences resulted from the lack of the primary SPI-2 regulator, SsrB. Interestingly, providing SsrAB *in trans* to these backgrounds caused a dramatic induction (∼50-fold) of *sseL* in *S. bongori* (to a much higher level than in *S.* Typhimurium), but only limited expression increase in *E. coli* (Fig. 5A), suggesting possible involvement of other *sseL* regulator(s), (see Discussion).

We further characterized *sseL* expression in *S. bongori* as an SsrB-negative host. A S. *bongori phoP* in-frame deletion mutant (DE.1.10.3) was constructed and the expression of *sseL::lacZ* was examined in the presence and absence of its native PhoP. In contrast to the reduced expression in the absence of PhoP in *S.* Typhimurium (SL1344 *ssrB* vs. SL1344 *phoP ssrB*; Fig. 2), no significant change in the expression of *sseL::lacZ* was found in the *S. bongori phoP* strain relative to the parental strain, and both expressed low levels of *sseL*. Nevertheless, providing *in trans* the *S.* Typhimurium PhoP (pPhoP_ST_), but not the *S. bongori* PhoP (pPhoP_SB_) resulted in elevated (>5-fold; *P*<0.0001) expression of *sseL* in the *S. bongori phoP* background (Fig. 5B). To confirm these results, using a *S.* Typhimurium host, we expressed *sseL::lacZ* in an SL1344 *phoP ssrB* strain complemented with either pPhoP_ST_ or pPhoP_SB_. Although the expression level following complementation was much lower in this background relative to *S. bongori* (further supporting the notion that an *sseL*-repressor might play a role in *S.* Typhimurium), higher levels of *sseL::lacZ* were constantly detected upon complementation with pPhoP_ST_ in comparison to pPhoP_SB_ (*P*<0.0001; Fig. 5C).

In agreement with the previously presented data, these results provided a further line of evidence that the *S.* Typhimurium PhoP can induce *sseL* expression independently of SsrB. However, unlike *S.* Typhimurium PhoP, the *S. bongori* ortholog did not seem to activate *sseL* expression.

Possible differences between the PhoP of *S. bongori* and *S. enterica* were intriguing. Comparing the sequence of PhoP from the *S. bongori* strain to its ortholog in *S.* Typhimurium (Fig. 5D) revealed a single amino acid difference at the C-terminal domain, harboring phenylalanine (F) instead of valine (V) at position 169. The C-terminal domain of the OmpR family members is thought to be involved in DNA-binding and protein-protein interactions with the RNA-polymerase (reviewed in [14]). The structure of the C- terminal domain of *E. coli* OmpR (OmpRc) has been determined (PDB ID: 1ODD) and was shown to compile 3 α-helices and 7 β-sheets [41]. Secondary structure prediction of the *Salmonella* PhoP using the SOPMA program [42] predicted very similar organization of the secondary elements and positioned residue 169V within the β5 strand (Figure 5D). To test whether V169F amino acid substitution in PhoP is responsible for the disability of the *S. bongori* strain to activate *sseL* expression, we engineered a construct harboring the *S. bongori phoPQ* sequence with a GTC (valine) codon instead of TTC (phenylalanine) at position 169 (pPhoP_SB169V_). Introducing pPhoP_SB169V_, but not the original pPhoP_SB_, into a *S. bongori phoP* strain elevated *sseL::lacZ* expression to a similar level that was found in the presence of the *S.* Typhimurium PhoP (pPhoP_ST_; Fig. 5B). We concluded from this analysis that the PhoP of the *S. bongori* strain was unable to activate *sseL* expression due to a single amino acid change at the C-terminal domain of this regulator.

### The PhoP of the *S. bongori* strain is impaired in activating the PhoPQ regulon

**Figure 6:**
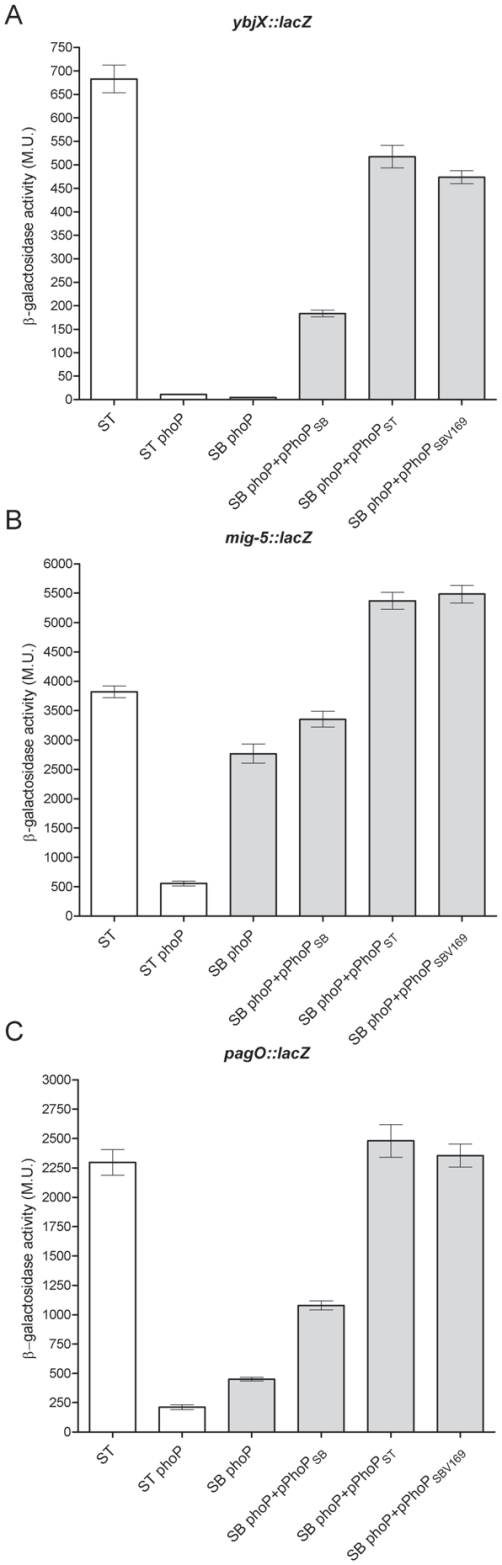
The PhoP from a *S. bongori* SARC 12 strain is impaired in regulating the *phoPQ* regulon. *S.* Typhimurium (ST), *S.* Typhimurium *phoP* (ST phoP), *S. bongori phoP* (SB phoP) and *S. bongori phoP* complemented with a *S. bongori phoPQ* (pPhoP_SB_), *S.* Typhimurium *phoPQ* (pPhoP_ST_), or the *S. bongori phoPQ* harboring V169 (pPhoP_SB169V_) expressing *ybjX::lacZ* (A), *mig-5::lacZ* (B) or *pagO::lacZ* (C) were examined for their β-galactosidase activity following growth under inducing conditions. The means with a standard error shown by the error bars are presented.

Next we asked whether the identified disparity in *S. bongori* PhoP affects its ability to activate other genes from the PhoP regulon, besides *sseL*. To answer that we investigated the expression of an ancestral (*ybjX*) and two *S.* Typhimurium-specific (*pagO* and *mig-5*) genes known to be under PhoP control, in *S.* Typhimurium and *S. bongori* hosts. As expected, this analysis showed significantly higher expression levels of *ybjX::lacZ*, *mig-5::lacZ* and *pagO::lacZ* in *S.* Typhimurium compared to a *S.* Typhimurium *phoP* strain. Up-regulation was observed when a *S. bongori phoP* strain was complemented with either *S.* Typhimurium PhoPQ (pPhoP_ST_) or with a *S. bongori* PhoPQ harboring V169 residue (pPhoP_SBV169_), but to a significantly lesser extent when it was complemented with the native *S. bongori* PhoPQ system (pPhoP_SB_; Figure 6). Taken together, these results suggested that the PhoP of the *S. bongori* strain is attenuated in activating expression of not only *sseL*, but also other members of the PhoP regulon, and that the valine residue at position 169 is required for the regulatory activity of PhoP.

## Discussion

Understanding the regulatory circuits governing the expression of virulence traits is important for a more complete understanding of bacterial pathogenesis and host-pathogen interactions. The virulence potential of many pathogens is greatly facilitated by the acquisition of new traits via lateral gene transfer. The ability of the recipient organism to benefit from these “imported goods” is largely dependent on their successful incorporation into a functional preexisting regulatory network.

Previous studies have shown a role for PhoP in controlling SPI-2 associated genes by indirect mechanisms. PhoP has been shown to bind the *ssrB* promoter and to affect SsrA levels post-transcriptionally [25]. In addition, PhoP affects the expression of SlyA [23]. Since SsrB and SlyA are both positive regulators of the SPI-2 regulon [22], [43], indirect effect by PhoP on SPI-2 gene expression occurs. Nonetheless, it seems that the functional integration of the PhoPQ pathway into the regulatory network governing SsrB-activated genes is even more convoluted. Here we elucidate another level of regulation and establish that the SPI-2 effector gene, *sseL*, is directly regulated by this ancestral regulator. Several lines of evidences indicated the ability of PhoP to promote expression of *sseL* in an SsrB-independent fashion: (i) reduced expression of *sseL::lacZ* was demonstrated in a *phoP ssrB* double mutant strain compared to the *ssrB* background in *S.* Typhimurium (Fig. 2A); (ii) diminution in the abundance of *sseL* transcripts in the *phoP ssrB* background in relation to the *ssrB* strain (Fig. 2B); (iii) a PhoP-mediated induction of *sseL::lacZ* in a *S. bongori* SsrB-free heterologous host (Fig. 5); (iv) the presence of two putative PhoP boxes in the promoter region of *sseL* (Fig. 3); and (v) direct *in-vitro* binding of His-PhoP to the promoter region of *sseL* in a gel mobility shift assay (Fig. 4). Collectively, our data suggest that PhoP directly activates *sseL* by a feed-forward regulatory mechanism. The feed-forward loop is a very efficient regulatory circuit [44], in which a transcription factor (PhoP) regulates a second transcription factor (SsrB) and both of them jointly control the expression of a third gene (*sseL*). This mode of regulation allows tuning up gene regulation and effective integration of different environmental signals sensed by PhoQ and SsrA. Controlling *sseL* expression by PhoP could be mediated either by transcription activation *per se* or by counteracting nucleoid-like proteins, such as H-NS, YdgT, and Hha that were shown to bind A+T rich sequences and repress transcription of SPI-2 genes (reviewed in [24]) including *sseL* specifically [9]. Such an activity of PhoP has been shown for *pagC*, in which PhoP counteracts H-NS silencing [45], [46].

Promoter analysis followed by site directed mutagenesis identified two putative PhoP binding sites in the promoter region of *sseL*, whereas one of them overlaps with the −10 element of *sseL*. Although this arrangement is not typical of PhoP dependent promoters, similar promoter architecture has been previously described. The PhoP-activated promoters of *ugtL* [47] and *iraP* [48] harbor two PhoP binding sites, and one of the sites in each promoter overlaps with the −10 element. Furthermore, it is now apparent that some PhoP-regulated promoters do not possess a canonical PhoP box in their PhoP binding sites. One of the PhoP-binding sites of *ugtL* and the *ssrB* promoters does not contain a PhoP box at all [25], [47]. Another interesting finding is that the second putative PhoP-binding site is downstream from the *sseL* site of transcriptional initiation. Although it is not very common among transcriptional regulators, accumulating evidences show that binding sites for transcriptional activators can be located downstream of the transcriptional initiation sites. For example, both the response regulator Rns from *E. coli* and SlyA from *Salmonella* have been shown to have binding sites downstream of the transcription start sites of target genes [47], [49]. Multiple binding sites for OmpR, which belongs to the same subfamily of response regulators as PhoP [50], were observed downstream of the transcriptional start sites of *ssrA* and *ssrB* in *Salmonella* [51].

Noteworthy, many of the PhoP-governed genes that do not have an orthodox PhoP promoter organization also require additional regulators to activate their transcription [29], including *ugtL* that requires SlyA in addition to PhoP [47]; and the *ssrB* promoter, which is autoregulated by SsrB [25]. These observations support the notion that the expression of some PhoP-regulated genes involves the function of PhoP in conjunction with other transcription regulators and that the promoter architectures of regulons such as PhoP or SsrB can be modular and evolvable over time [52].

The possibility that *sseL* expression requires the coordinated activity of several regulators is also supported by comparing its expression in *E. coli* and *S. bongori* in the presence of SsrB. Introducing SsrAB into these hosts led to a profound expression of *sseL::lacZ* in *S. bongori*, but to only moderate levels in *E. coli* (Fig. 5A). These results suggest that certain *Salmonella* spp. factors, which are not present (or at least different enough) in *E. coli*, are still required for maximal *sseL* expression, in conjunction with SsrB. Similarly, introducing SsrB or PhoP into *S. bongori* resulted in a much higher induction of *sseL* expression than in *S.* Typhimurium background (Fig. 5), implying the possibility that a particular *sseL*-negative regulator, not present in *S. bongori*, may play a role in *S.* Typhimurium.

Surprisingly, as opposed to the *S.* Typhimurium PhoP, an impaired ortholog from a *S. bongori* SARC 12 strain was not able to activate *sseL* transcription. We showed that this distinction resulted from a single amino acid variation at position 169, containing phenylalanine in place of valine (Fig. 5D), that was important for the expression of *sseL* as well as for other PhoP-regulated genes (Fig. 5 and Fig. 6). A secondary structure prediction of *Salmonella* PhoP and comparison with the crystal structure of OmpRc positioned V169 within the β5 domain of the PhoP C-terminus region. A mutation at the equivalent position (Leu 175) was identified in the VirG regulator (also belonging to the OmpR family) and demonstrated to be part of a conserved internal hydrophobic core, important for stabilizing protein conformation [14], [53]. Hydrophobic core residues are highly conserved across the OmpR family members, which presumed to share a common fold [14]. Based on that, we believe that the V to F substitution at this position changes protein conformation and therefore interferes with the regulatory activity of PhoP in *S. bongori*. It is worth pointing out that PhoP from *E. coli* K-12 (accession number BAA35952) and other *S. bongori* strains (ABG34164, ABG34169) harbor a V169 residue, suggesting that this missense mutation in the SARC 12 strain probably arose relatively recently.

Collectively, the experimental setup described here demonstrates an interesting interface between the SsrB and the PhoP pathways and shows the evolvement of a feed-forward regulatory loop to control newly acquired virulence genes. The interaction between the conserved housekeeping regulator PhoP and a horizontally acquired SsrB-regulated gene not only provides an interesting example for functional integration of incoming genes into the core regulatory network of a pathogen, but also reveals dynamics and plasticity in the evolution of regulatory circuits.

It is tempting to hypothesize that PhoP contributes directly to the expression of other SPI-2 effector genes as well. Previously we showed that expression of the effectors SseK1 and SseK2 was significantly reduced under SPI-2 induction conditions in the absence of PhoP [54]. This observation, together with the presence of PhoP-box resembling sequences in the promoter regions of *sseK1* and *sseK2* (data not shown), is in agreement with this idea.

The acquisition of new traits and their regulatory assimilation are central to the ability of prokaryotes to evolve novel phenotypes, adapting to and occupying new niches. Coordination of laterally acquired virulence genes by several systems is expected to contribute to fine-tuning of a *Salmonella* intracellular virulence program and provide more flexibility and sensitivity in its response to the host milieu.

## Materials and Methods

### Bacterial strains and *in vitro* growth conditions

Bacterial strains and plasmids utilized in this study are listed in Table 1. *S.* Typhimurium SL1344 and *S. bongori* SARC 12 (a generous gift from Prof. M. Hensel) were used as the wild-type strains. *S.* Typhimurium *phoP ssrB* (OG2011) double mutant strain was generated by P22 transduction of *phoP::kan* into SL1344 *ssrB* background. A spontaneous streptomycin-resistant *S. bongori* SARC 12 strain was isolated on LB agar plates containing 100 µg ml^−1^ streptomycin and was used as the parental strain for a *S. bongori phoP* mutant (DE1.10.3). DE1.10.3 carries an in-frame deletion (amino acids 8–217) of *phoP* and was generated by allelic exchange using the counter-selectable suicide vector pRE112 [55] as previously described [56].

**Table 1:** Bacterial strains and plasmids used in the study.

*Strain or plasmid*	*Genotype and description* *	*Reference or source*
***Salmonella***		
*S.* TyphimuriumSL1344	wild-type Sm^r^ *xyl hisG rpsL*	ATCC
OG2011 (*phoP ssrB*)	SL1344 *phoP*::Tn10 Tc^r^ *ssrB*::kan transduced by P22 from *ssrB*	This study
*phoP*	SL1344 *phoP*::Tn10 Tc^r^	[58]
*ssrB*	SL1344 *ssrB*::kan transduced by P22 from 14028s	[59]
*S. bongori* SARC 12		[60]M. Hensel lab
DE1.10.3 *(phoP)*	*S. bongori* SARC12Δ *phoP*	This study
***E. coli***		
BL21(DE3)	F- *omp*T *hsd*S_β_(r_β_-m_β_-) *dcm gal* (DE3) *tonA*	Lab collection
DH5α	F- ϕ80*lac*ZΔM15 Δ(*lac*ZYA-*arg*F)U169 *deo*R *rec*A1 *end*A1 *hsd*R17(rk^−^, mk^+^) *sup*E44 *thi*-1 *gyr*A96 *rel*A1 λ-	Lab collection
SM10 λ*pir*	*thi thr leu tonA lacY supE recA:*:RP4-2-Tc::Mu Km λ*pir*	Lab collection
TOP10	*mcrA* Δ(*mrr*-*hsdRMS*-*mcrBC*) ϕ80*lacZ* ΔM15 Δ*lacX*74 *deoR recA*1*araD*139 Δ(*ara*, *leu*) 7697 *galU galK* λ- *rpsL endA*1 *mup*G	Invitrogen
**Plasmids**		
pACYC184	Tc^r^ Cm^r^ cloning vector	NEB
pCR-Blunt	Kan^r^ cloning vector	Invitrogen
pDE-PhoPQ_SB_	*S. bongori phoPQ* cloned into pACYC184	This study
pDE-PhoPQ_SB169V_	*S. bongori phoP* haboring V169 cloned into pACYC184	This study
pDE-PhoPQ_ST_	*S.* Typhimurium *phoPQ* cloned into pACYC184	This study
pDE-SsrAB_ST_	*S.* Typhimurium *ssrAB* cloned into pACYC184	This study
pET-28a(+)	Kan^r^ cloning vector for N-terminal His tag fusions	Novagen
pRE112	pGP704 suicide plasmid; *pir* dependent; *sacB*	[55]
pMC1403	Amp^r^ *lacZY* cloning vector	[30]
pOG-His-PhoP	N-terminus 6-His tag of PhoP in pET28-a	This study
pOG-pagO-lacZ	*pagO* fused to *lacZ* in pMC1403	This study
pOG-mig5-lacZ	*fkpA* fused to *lacZ* in pMC1403	This study
pOG-PCR-sseL-RACE	RLM-RACE of *sseL* cloned into pCR-Blunt	This study
pOG-sseL-lacZ	*sseL* fused to *lacZ* in pMC1403	This study
pOG-sseL-lacZ-S1	S1 mutation in *sseL* promoter fused to *lacZ* in pMC1403	This study
pOG-sseL-lacZ-S2	S2 mutation in *sseL* promoter fused to *lacZ* in pMC1403	This study
pOG-sseL-lacZ-S1+2	S1+S2 mutations in *sseL* promoter fused to *lacZ* in pMC1403	This study
pOG-ybjX-lacZ	*ybjX* fused to *lacZ* in pMC1403	This study
pUC19	Amp^r^ high-copy number cloning vector	NEB

*Sm, streptomycin; Cm, chloramphenicol; Kan, kanamycin.

Bacterial cultures were routinely maintained in Luria-Bertani (LB) liquid medium or on LB agar plates supplemented with the appropriate antibiotic(s) at the following concentrations: chloramphenicol, 25 µg ml^−1^; kanamycin, 50 µg ml^−1^; ampicillin, 100 µg ml^−1^; and streptomycin, 100 µg ml^−1^. To examine expression under SPI-2 induction conditions, *Salmonella* strains were grown in low phosphate low magnesium minimal (LPM) medium containing 80 mM MES (pH 5.8), 5 mM KCl, 7.5 mM (NH_4_)SO_4_, 0.5 mM K_2_SO_4_, 337 µM K_2_HPO_4_/KH_2_PO_4_ (pH 7.4), 20 mM MgCl_2_, 38 mM glycerol, and 0.1% Casamino acids [57] with aeration at 37°C.

### Construction of *lacZ* reporter fusions

PCR fragments containing ∼500-bp from the upstream region and the first seven amino acid codons of *sseL*, *mig-5*, *pagO*, and *ybjX* were amplified using the primers OG-140 and OG-141; OG-132 and OG-133; OG-128 and OG-129; OG-130 and OG-131, respectively. PCR products were cloned into pCR-Blunt, and moved into pMC1403 digested with *Eco*RI and *Bam*HI, resulting in pOG-sseL::lacZ, pOG-mig-5::lacZ, pOG-pagO::lacZ, and pOG-ybjX::lacZ.

To generate an *sseL* promoter harboring nucleotides substitution in the putative PhoP box 1, 325-bp and 593-bp PCR products were amplified using pOG-sseL::lacZ as template and the primer pairs OG-171 and OG-185, and OG-167 and OG-172, respectively. The resulting PCR products were used as template for a secondary PCR reaction using the primers OG-167 and OG-185. The obtained 895-bp PCR product was cloned using *Eco*RI and *Bam*HI into pMC1403, generating pOG-sseL::lacZ-S1. Similarly, the primers pairs OG-169, OG-185 and OG-170, OG-167, were used to amplify 360-bp and 556-bp PCR products, respectively, that were used to create a parallel mutation in PhoP box 2. The obtained PCR fragment was cloned as described above into pMC1403, generating pOG-sseL::lacZ -S2. To generate an *sseL::lacZ* promoter harboring both S1 and S2 mutations, we used pOG-sseL::lacZ-S1 as a template in PCR reactions with primers OG-167, OG-170 and OG-169, OG-185. The resulting PCR products were used as a template for a subsequent PCR amplification with primer OG-167 and OG-185, and the obtained 895-bp PCR product was cloned into pMC1403, generating pOG-sseL::lacZ-S1+2. All the final plasmids described above were verified by DNA sequencing.

### Construction of PhoPQ and SsrAB complementation constructs

A 4802-bp PCR fragment corresponding to the *S.* Typhimurium or *S. bongori phoPQ* was amplified using oligonucleotides DE-12 and DE-13 containing *Xba*I and *Sph*I sites, respectively. The digested fragment was cloned into pACYC184, generating pDE-PhoPQ_ST_ or pDE-PhoPQ_SB_. Similarly, the primers DE-23 and DE-24 were used to amplify a 5610–bp DNA fragment containing the *ssrAB* operon. The resulting PCR product was cloned into pACYC184, resulting in the plasmid pDE-SsrAB. To generate a construct harboring a *S. bongori* PhoP with V residue at position 169, 959-bp and 2108-bp PCR products were amplified using pDE-PhoPQ_SB_ as template and the primer pairs DE-11, DE-101; and DE-12, DE-102, respectively. The resulting PCR products were used as a template for a secondary PCR reaction using the primers DE-11 and DE-12. The obtained 3067-bp PCR product was cloned using *Pst*I and *Sph*I into pDE-PhoPQ_SB_, generating pDE-PhoPQ-_SB169V_.

### β-galactosidase assays *in-vitro*

β-galactosidase assays were performed according to [61]. *Salmonella* strains were grown in LB or in LPM medium. The assays were performed with 100 µl of culture, and the substrate for β-galactosidase hydrolysis was *o*-nitrophenyl-β-D-galactopyranoside (ONPG, Sigma).

### β-galactosidase assays in macrophages

1 ml of an overnight culture grown in LB+Amp was harvested and resuspended in 1 ml of DMEM. 100 µl of the suspended bacteria were used to inoculate each well of a six-well plate containing J774.1 mouse macrophages at a multiplicity of infection of 100. Plates were centrifuged at 500 *g* for 5 min and incubated at 37°C/5% CO_2_ for 20 min to allow adherence. Infected cells were washed three times with PBS, 2 ml DMEM containing 50 µg/ml gentamicin was added to each well, and the plates were incubated at 37°C/5% CO_2_. After 6 h, macrophages were washed with PBS, scraped from the plates, centrifuged, and resuspended in 115 µl of PBS. 15 µl of the suspended cells was added to 135 µl of PBS buffer containing 1% Triton X- 100 and 0.1% SDS. Serial dilutions were plated on LB to determine intracellular bacterial count. The remaining 100 µl of the cell suspension was directly used for β-galactosidase assay by adding 0.9 ml of Z buffer, three drops of chloroform, and two drops of 0.1% SDS followed by vortexing. An aliquot of 200 µl of ONPG (4 mg/ml) was added and reaction was stopped by the addition of 0.5 ml of 1 M Na_2_CO_3_. Following centrifugation, optical density of the supernatant was measured at 420 nm. The β-galactosidase activity was calculated according to: OD_420_/[*t* (min)×volume (ml)], and was normalized according to the intracellular bacterial count.

### Quantitative real-time PCR analysis

*Salmonella* RNA was extracted from late-exponential phase cultures that were grown in LPM medium for 6 h using the Qiagen RNAprotect Bacteria Reagent and the RNeasy mini kit according to the manufacturer's instructions, including an on-column DNase digest using the RNase-free DNase set (Qiagen). The quantity and quality of the extracted RNA were determined by an ND-1000 spectrophotometer (NanoDrop Technologies). To diminish any genomic DNA contamination, RNA was secondarily treated with an RNase-free DNase I (Invitrogen). 0.5 µg of DNase I-treated RNA was subjected to a first strand cDNA synthesis using the QuantiTect Reverse Transcription Kit (Qiagen). Real-time PCR reactions were performed in an Applied Biosystems 7500 Fast Real-time PCR System. Each reaction was carried out in a total volume of 10 µl on a 96-well optical reaction plate (Applied Biosystems) containing 5 µl FastStart Universal SYBR Green Master (ROX) mix (Roche Applied Science), 1 µl cDNA, and two gene-specific primers at a final concentration of 0.3 µM each. Real-time cycling conditions were as follows: 50°C for 2 min; 95°C for 10 min; and 40 cycles of 95°C for 15 s, 60°C for 1 min. No-template and no reverse-transcriptase controls were included for each primer set and template. Melting curve analysis verified that each reaction contained a single PCR product. Relative quantification of *sseL* transcripts was evaluated using the comparative C_t_ method. The housekeeping gene, *rpoD*, was used as the endogenous normalization control. The ΔC_t_ values were calculated by determining the difference in threshold values for *sseL* and *rpoD* in the wild-type vs. the mutant strains. Calculation of ΔΔC_t_ involved the subtraction of the normalized wild-type ΔC_t_ value from the normalized ΔC_t_ value of the compared mutant. Fold-differences in gene expression were calculated as 2^−ΔΔCT^.

Gene-specific primers were designed using PRIMER3 software (http://primer3.sourceforge.net/), are listed in Table 2, and correspond to the following genes: *rpoD*, OG-220 and OG-221; *sseL*, OG-233 and OG-234.

**Table 2:** List of primers used in the study.

*Primer*	*Sequence (5′-3′)*
DE-11	TGATCGCTTATCAGTCCACCC
DE-12	TTTGCATGCACATCATACTGATCCAGATG
DE-13	TTTTCTAGATGTCGATGGACGCTACGGCG
DE-23	TTTGCATGCAATACTGCGTGGCGTAAGGC
DE-24	TTT TCTAGAATGTAGCTGTTATCAATGGGC
DE-101	AGCGAATCTTTG CTGACAACTTTACCAT
DE-102	GTTGTCAGCAAAGATTCGCTGATGCT
OG-128	ACG**GAATTC**AGCCGGTGCTGTTTCTGGGGC
OG-129	TTT**GGATCC**ATAGATATCGACACCTTGCGC
OG-130	TTT**GAATTC**CCTCTTTTATCTGGTTCTCCGCC
OG-131	TTT**GGATCC**GTCATGATCGTAATCCGCGAC
OG-132	TTT**GAATTC**CATAACTGTGACGCGACAACCGG
OG-133	TTT**GGATCC**TGTGCTGGTTGGTTTTGTTCC
OG-140	TTT**GAATTC**CCCCATTACGCTACTGGCCC
OG-141	TTT**GGATCC**AATGTAAGCGCCTCATCGCTCACC
OG-146	AAA**CATATG**ATGCGCGTACTGGTTGTAG
OG-147	AAA**GGATCC**GCGAGCAAATTTATTCATTAGC
OG-167	GTTCCGCGCACATTTCCCCG
OG-169	TTCGCTACT**GG**CACTTACCAGGAAACAGAGC
OG-170	TTCCTGGTAAGTG**CC**AGTAGCGAATATGATGTAATG
OG-171	GAATATATGTGTAAATTCAC**GG**TACCG
OG-172	GTA**CC**GTGAATTTACACATATATTCATTTTGC
OG-185	ACGACAGTATCGGCCTCAGG
OG-220	GTGAAATGGGCACTGTTGAACTG
OG-221	TTCCAGCAGATAGGTAATGGCTTC
OG-233	AGTTCGCTCAGACAGATCAAG
OG-234	AGGATGAATCAGCCCAATAGG
OG-239	AATTATACATTACAT CATATTCGC
OG-240	CGCTCACCTCTTCTGTATATA
OG-245	GCGGAACCTGCCATATAAAG
OG-246	CGACAACATTCGCGCACCAC

### 5′ RLM-RACE

RNA ligase-mediated rapid amplification of cDNA ends (RLM-RACE) was used to determine the transcription start site of *sseL*. Total RNA was extracted from *S.* Typhimurium SL1344 culture grown to late exponential phase in LPM medium under SPI-2 induction conditions. Rapid amplification of 5′ cDNA ends was carried out using a FirstChoice RLM-RACE kit (Ambion) according to the manufacturer's instructions, excluding a CIP treatment. Briefly, 300 ng of *Salmonella* extracted RNA was subjected to a Tabacco Acid Pyrophosphatase treatment followed by 5′ RACE adapter ligation and reverse transcription reactions. The nested-PCR conditions for 5′ outer PCR were 1 µl from the RT reaction, 10 pmol gene-specific outer primer OG-246, 10 pmol 5′ RACE outer primer (Ambion), 2.5 units of PfuTurbo DNA polymerase (Stratagene), 5 µl of 10× PfuTurbo PCR buffer, 4 µl of dNTP mix, and 35 µl of H_2_O. The PCR conditions were as follows: 1 cycle of 95°C for 5 min; 35 cycles of 94°C for 30 s, 60°C for 30 s, and 72°C for 60 s; and 1 cycle of 72°C for 10 min. The 5′ RACE inner PCR reaction was carried out with 10 pmol gene-specific inner primer OG-245 and 10 pmol 5′ RACE inner primer (Ambion) using the products generated with the outer primer set as a template. The same conditions as for the 5′ outer PCR were used, but the annealing temperature was 53°C. The resulting PCR products were analyzed on 2% agarose gels, purified, and then cloned into pCR-Blunt (Invitrogen). Four individual clones were sequenced to determine the transcription initiation site of *sseL*.

### Construction of *S.* Typhimurium His tagged PhoP

A 706-bp fragment containing the *S.* Typhimurium *phoP* gene was amplified by PCR using the primers OG-146 and OG-147. The resulting PCR fragment was cloned into pCR-Blunt, generating pOG-PCR-phoP. The latter was digested with *Nde*I and *Bam*HI, and the resulting insert was cloned into the pET-28a vector (Novagen) digested with the same enzymes resulting in the plasmid pOG-His-PhoP. This plasmid expresses full-length *S.* Typhimurium PhoP fused to a His_6_ tag on the N terminus, with a predicted molecular mass of 27.8 kDa, under the control of the T7 promoter.

### Over expression of His-tagged PhoP and protein purification

*E. coli* BL-21 (DE3) strain carrying pOG-His-PhoP was grown overnight in LB medium containing 50 µg/ml Kan at 37°C with vigorous aeration. The culture was diluted 1∶200 into 300 ml of fresh LB and grown for 2.5 h. To induce expression of the recombinant protein, IPTG was added to 1 mM final concentration, and the culture was grown for an additional 3 h at 30°C and harvested by centrifugation. The pellet was resuspended in 10 ml lysis buffer [50 mM NaH_2_PO_4_, 300 mM NaCl, 10 mM imidazole, 1 mM MgCl_2_ in the presence of DNase I (5 µg/ml) and RNase A (10 µg/ml)]. Cells were disrupted using a French Press (three passages, 1000 PSI) and then centrifuged at 20,000 rpm for 30 min. His_6_-PhoP was purified from 1 ml of the soluble fraction by a nickel-affinity chromatography using ÄKTApurifier FPLC system (GE Healthcare) and a HisTrap FF 1 ml column (GE Healthcare) at 4°C. The column was washed with 5 ml of washing buffer (50 mM NaH_2_PO_4_, 300 mM NaCl, 20 mM imidazole and 1 mM MgCl_2_). His_6_-PhoP was eluted with 5 ml elution buffer (50 mM NaH_2_PO_4_, 300 mM NaCl, 250 mM imidazole and 1 mM MgCl_2_) and analyzed on an SDS-polyacrylamide gel. The selected elute fractions were then collected and dialyzed against dialysis buffer [20 mM HEPES (pH 8.0), 100 mM KCl, 20% glycerol, 1 mM DTT and 1 mM MgCl_2_] on a Sephacryl S-200 gel filtration column. Dialyzed fractions were analyzed by UV absorbance at 280 nm and on an SDS-polyacrylamide gel. The appropriate fractions were pooled and concentrated using a 10 kDa Amicon Ultra centrifugal filter devices (Millipore). Protein samples were stored at −80°C until use.

### Mobility shift assay

Gel retardation assays were carried out using the Dig Gel Shift Kit (Roche). A 128-bp fragment corresponding to the upstream regulatory sequences of *sseL* was generated using the primers OG-239 and OG-240 and labeled with digoxigenin (DIG)-dUTP mediated by terminal transferase (Roche) according to the manufacturer's instructions. Purified His-PhoP was incubated at 37°C for 30 min with 50 mM acetyl phosphate in a 20 µl reaction buffer containing 20 mM HEPES (pH 7.6), 1 mM EDTA, 10 mM (NH_4_)_2_SO_4_, 5 mM DTT, 0.2% (w/v) Tween 20, 30 mM KCl, and 0.1 µg Poly L-lysine in the presence of 1 µg poly (dI-dc), an unspecific DNA competitor. 15 fmol of the labeled probe was added, and the DNA-protein mixtures were incubated for a further 30 min at 37°C. The binding reactions were stopped by the addition of 5 µl of loading dye and were immediately resolved on a 6% nondenaturing polyacrylamide gel in 0.5× Tris-glycine running buffer (25 mM Tris, 250 mM Glycine; pH 8.3) at 100 V. Subsequently, the gels were electroblotted onto a positively charged nylon membrane (Hybond-N+, Amersham Biosciences) in 0.5× TBE buffer at 400 mA for 1 h and fixed by UV cross-linking. Detection of the DIG-labeled DNA probes by anti-DIG Fab fragment-alkaline phosphatase conjugate (Roche) in the presence of CSPD substrate was performed according to the manufacturer's instructions.

### Statistical analysis

Data of the β-galactosidase and the RT-PCR assays are expressed as mean ± standard error. The statistical significance between different values was calculated by the unpaired *t*-test with two-tailed P value. *P*<0.05 was considered to be statistically significant.
